# Impact of the SARS‐CoV‐2 pandemic on the overall respiratory viruses' transmission in a cancer care setting

**DOI:** 10.1002/iid3.1073

**Published:** 2023-11-22

**Authors:** Sawsan Mubarak, Osama Alsmadi, Abdelghani Tbakhi, Osama Abu Ata, Ala'a Hassan, Hadeel AlGhawrie

**Affiliations:** ^1^ Infection Control Program King Hussein Cancer Center Amman Jordan; ^2^ Department of Cell Therapy & Applied Genomics King Hussein Cancer Center Amman Jordan; ^3^ Department of Internal Medicine King Hussein Cancer Center Amman Jordan

**Keywords:** COVID‐19, flu viruses, joint point regression, respiratory viruses

## Abstract

**Introduction and Objective:**

The emergence of the COVID‐19 pandemic raised questions about the interaction between severe acute respiratory syndrome coronavirus 2 (SARS‐CoV‐2) and other respiratory viruses. The objective of this study is to validate the impact of the SARS‐CoV‐2 pandemic and its interventional measures on the respiratory viruses' transmission/infection rates.

**Methods:**

A retrospective chart review was conducted for cancer patients who underwent laboratory‐confirmed respiratory virus polymerase chain reaction (PCR) testing from January 2018 to June 2022. COVID‐19 PCR tests from March 2020 to June 2022 were also included. Joinpoint regression analysis was applied to evaluate trends in respiratory virus rates. Statistical analysis was performed using Statistical Package for Social Science software.

**Results:**

A total of 6298 respiratory virus PCRs and 40,000 COVID‐19 PCRs were performed. Data showed a significant decrease in respiratory viruses' positive cases, total respiratory tests, and respiratory viruses' activity during the pandemic period compared with the pre‐pandemic period (*p* = .0209, .026, and .028, respectively). The joinpoint regression analysis showed a significant decrease of 13.85% in the tested positive cases of respiratory viruses between the years 2018 and 2022. Monthly, the analysis indicated a significant decrease in the positive cases by 13.46% from December 2019 to May 2021. Weekly analysis following lockdown initiation showed a reduction in respiratory virus cases.

**Conclusion:**

This study provides valuable insights into the interplay between COVID‐19 and other respiratory viruses, suggesting that the measures taken for COVID‐19 were effective in reducing the spread of viral respiratory infections, aiding future infection control strategies to protect vulnerable populations, including cancer patients, from seasonal respiratory infections.

## Graphical abstract

This study investigates the impact of the COVID‐19 pandemic on respiratory virus transmission rates, particularly in cancer patients. Through retrospective analysis of polymerase chain reaction testing data, significant reductions in positive cases and overall activity of respiratory viruses during the pandemic were observed. The findings highlight the effectiveness of COVID‐19 measures in curbing the spread of respiratory infections, offering insights for future infection control strategies, especially for vulnerable populations.

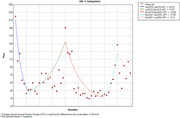

## 1 INTRODUCTION

Influenza is a respiratory illness that affects the respiratory tract, which may be limited to the upper respiratory tract, however, it spreads to the lower respiratory tract and causes respiratory failure.^1^ Influenza is recognized as a global public health concern, impacting around 5%–10% of the population resulting in a high number of fatalities ranging from 250,000 to 500,000 deaths each year.^2^ A study from 2017 showed a substantially higher mortality burden of 290,000–650,000 influenza‐associated deaths from respiratory infections alone.^3^ Another study in early 2019 published by the Global Burden of Disease Study estimated a range of 99,000–200,000 annual deaths from lower respiratory tract infections directly attributable to influenza viruses.^4^

Seasonal influenza usually occurs as annual outbreaks, but the timing of the onset peak, and end of activity varies among different regions worldwide. In Jordan, similar to other countries in the Northern Hemisphere, epidemics of influenza typically occur during the autumn and peak during the winter season.^5^

In patients who are immunocompromised due to an underlying disease, influenza infection can cause significant morbidity and mortality.^6^ Cancer patients are more likely to develop influenza complications from upper and lower respiratory tract infections. Oncology patients have a three to five times greater probability of hospitalization due to influenza infections compared to the general population, with a mortality rate of 9% (relative risk of four compared to the general population).^1^, ^7^ Each year, 441 per 100,000 oncology patients are hospitalized in the United States because of influenza virus infections which are three to five times greater than the average for the population. Moreover, the mortality rate is 9% in oncology patients.^7^

In December 2019, the city of Wuhan, China, announced the first incidence of pneumonia cases in the city, which was quickly followed by a global widespread.^8^ The coronavirus infection 2019 has emerged in many countries since December 2019 and was referred to as COVID‐19. COVID‐19 disease is caused by severe acute respiratory syndrome coronavirus 2 (SARS‐CoV‐2) is responsible for the outbreak that led to a global public health crisis.^9^

The Mortality and Morbidity Weekly Report (MMWR) by the Center for Disease Control and Prevention (CDC) showed a decline in the testing of flu viruses' activity (percentage of respiratory specimens submitted for influenza testing that yielded positive results) during the COVID‐19 pandemic in the United States, Australia, Chile, and South Africa, 2020.^10^ The observed decline was attributed to artifact changes related to hospitals seeking protective behaviors for respiratory illnesses and real changes related to a reduction in circulating respiratory viruses through the implementation of nonpharmaceutical interventions against COVID‐19.^11^ In 2020–2021, COVID‐19 mitigation techniques such as wearing face masks, staying at home, hand washing, school closures, reduced travel, increased ventilation of indoor spaces, and avoiding the crowded places, close contacts, and closed spaces (3Cs) and physical isolation were expected to have contributed to a drop in flu incidence, hospitalizations, and deaths. During the 2020–2021 flu season, vaccination against influenza may have resulted in a reduction in flu illnesses.^11^, ^12^

In the United States, the 2020–2021 influenza season has been characterized by an unusually low circulation of influenza viruses in the United States. From September 2020 through the week ending May 15, 2021, reflected just 0.051% positivity of respiratory specimens tested and reported to the US Centers for Disease Control and Prevention for influenza viruses at US public health laboratories, and 0.18% positivity at US clinical laboratories (compared with 10%–19% in recent years), this season's low flu activity resulted in much fewer flu infections, hospitalizations, and fatalities.^13^ In Singapore, COVID‐19 pandemic control efforts were linked to alterations in a wide spectrum of respiratory viruses, with the most significant changes occurring during the lockdown. The patterns of decline and subsequent reemergence, on the other hand, varied between viruses and pandemic reaction phases.^14^ Another report revealed that patients who were coinfected with SARS‐CoV‐2 and influenza B virus have a higher risk of developing poor outcomes, so the detection of both viruses was recommended during the COVID‐19 outbreak.^6^

There is currently a debate about SARS‐CoV‐2 seasonality and its impact on seasonal respiratory viruses, as epidemiological data are scarce. Analysis of this dataset provides a comprehensive assessment of the impact of the COVID‐19 pandemic on the circulation of seasonal respiratory viruses. To the best of our knowledge, this is the first study in Jordan to describe the concurrent epidemiology of seasonal respiratory viruses and SARS‐CoV‐2 during the 2020–2021 season. This study investigates the impact of the SARS‐CoV‐2 pandemic on influenza infection and overall respiratory virus transmission in a cancer care setting at King Hussein Cancer Center (KHCC). This could offer available information to the infection control team about the effectiveness of the precautions that were decided for COVID‐19 in decreasing the seasonal respiratory viruses. This may encourage health leaders in the future to adopt more infection control precautions to protect patients with cancer from the seasonal flu and other respiratory infections.

### 1.1 Study objective

This study aims to validate the impact of the SARS‐CoV‐2 pandemic and its interventional measures on the respiratory viruses' transmission and infection rates, in addition, to assess the respiratory viral infection prevalence rates during and before the SARS‐CoV‐2 pandemic among patients at KHCC between the years (2018–2022).

## 2 METHOD

This is a retrospective study designed by chart review of patients at King Hussein Cancer Center. All cancer patients (pediatrics and adults) who had respiratory symptoms and were diagnosed by laboratory‐confirmed Respiratory virus polymerase chain reaction (PCR) testing from January 2018 to June 2022 at KHCC were included in the study, in addition to that, all COVID‐19 PCR tests done from March 2020 to June 2022 were included in the study.

The Respiratory Viral Panel detects influenza virus, rhinoviruses, coronaviruses (other than COVID‐19), respiratory syncytial virus (RSV), parainfluenza viruses (1, 2, 3, and 4), adenoviruses, human metapneumovirus, and enteroviruses.

Data were categorized into prepandemic (before March 21, 2020) and pandemic (March 21, 2020) periods. On March 20, the Jordanian government announced plans for a national lockdown. Respiratory virus activity was measured as the ratio of positive cases to the total tests performed. Data were analyzed using the Statistical Package for Social Science software (IBM) to calculate case frequency and respiratory virus activity and report the difference in respiratory viruses' activity before and after the pandemic. We used a paired *t*‐test with a significance level of *p* = .05.

Parallel time series were constructed for all respiratory virus positive cases as time‐dependent variables recorded between January 2018 and June 2022. Joinpoint Regression Software (4.3.1.0, the Surveillance Research Program; US NCI) was adopted to break the time series into linear subtrends and to identify the number and place of the joinpoints that represent the moments of trend change; the estimated annual percentage change (APC) of the dependent variable for each time segment; and the average annual percentage change in the whole period studied. The software takes trend data and fits the simplest joinpoint model that the data allows. The user supplies the minimum and maximum number of joinpoints. The program starts with the minimum number of joinpoints (e.g., 0 joinpoints, which is a straight line) and tests whether more joinpoints are statistically significant and must be added to the model (up to that maximum number). This enables the user to test whether an apparent change in trend is statistically significant. The APCs of the indicators were calculated from 2018 to 2022, based on the model of regression by joinpoint regression. The *p* values of the slope changes of APCs are calculated from the *t*‐test based on asymptotic normality. Two‐sided *t*‐tests at *p* < .05 were used to test whether APCs were statistically significantly different from zero; 95% confidence intervals for each segment were calculated.

## 3 RESULTS

From January 2018 to June 2022, 6,298 respiratory virus PCRs were performed, and more than 40,000 COVID‐19 PCR tests were performed during the pandemic period until June 2022. The cumulative results of respiratory virus tests and COVID‐19 tests are shown in Table 1.

**Table 1:** Cumulative results of respiratory viruses' tests and COVID‐19 tests.

		Respiratory viruses'				COVID		
Year	Positive	Negative	Total	Viruses activity	Positive	Negative	Total	Viruses activity
2018	795	844	1639	48.51%				
2019	818	379	1197	68.34%				
2020	582	1125	1707	34.09%	769	9056	9825	7.83%
2021	531	347	878	60.48%	991	21027	22018	4.50%
2022	466	411	877	53.14%	1146	8027	9173	12.49%

Table 1 presents the cumulative outcomes of respiratory virus tests and COVID‐19 tests over a span of 5 years. The data is categorized into distinct years, with corresponding information regarding positive and negative results for each test type. The findings reveal fluctuations in viruses' activity and testing outcomes across the specified years. In 2018 and 2019, the virus activity for respiratory viruses was 48.51% and 68.34% respectively. The subsequent year, 2020, saw a decrease in respiratory virus activity to 34.09%. In 2021, respiratory viruses' activity increased to 60.48%. Finally, in 2022, respiratory viruses' activity reached 53.14%. The observed trends highlight the varying impact of respiratory viruses and COVID‐19 across different years, with notable changes in viruses' activity and testing outcomes. These shifts provide insights into the epidemiological dynamics of these respiratory infections within the specified time frame.

Table 2 presents a comparative analysis between the results obtained during the pandemic period and the prepandemic period. The data is structured to demonstrate the monthly mean cases for three key parameters: positive cases, total tests conducted, and respiratory viruses' activity. Notably, the monthly mean positive cases were lower during the pandemic phase (46 cases) compared to the prepandemic phase (71 cases), with a statistically significant difference supported by a *p* value of .0209. Likewise, total tests exhibited a decrease during the pandemic period (94 tests on average) in comparison to the pre‐pandemic period (139 tests on average). This difference is statistically significant with a *p* value of .026.

**Table 2:** Results of respiratory viruses during the pandemic period compared with the prepandemic period.

Monthly mean cases	Prepandemic	Pandemic	*p* Values
Positive cases	71	46	.0209^a^
Total tests	139	94	.026^b^
Respiratory viruses' activity	57%	48%	.028^c^

*Note*: A *t*‐test for two independent means was done.

^a^ *t*‐Test was done for the positive cases prepandemic and during the pandemic.

^b^ *t*‐Test was done for the total tests prepandemic and during the pandemic.

^c^ *t*‐Test was done for respiratory viruses' activity prepandemic and during the pandemic.

**Figure 1:**
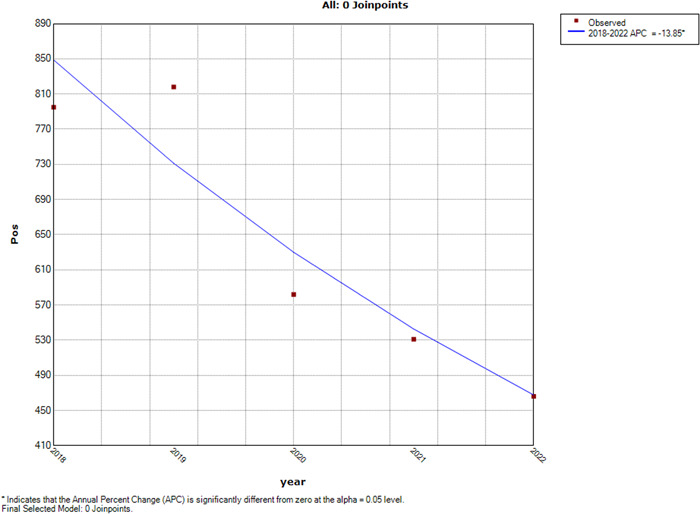
Joinpoint analysis of changes in respiratory viruses' positive cases per year.

The incidences of respiratory virus cases between January 2018 and June 2022 are shown in Figure 1. The results of the joinpoint regression analysis revealed a substantial decrease in the frequency of tested‐positive cases of respiratory viruses'. Specifically, we observed a remarkable reduction of 13.85% in these positive cases. This reduction was statistically significant, highlighting a clear and notable change in the landscape of respiratory virus incidence over the study duration. This decline in tested‐positive cases of respiratory viruses underscores a meaningful shift in the prevalence of the disease over the examined period.

**Figure 2:**
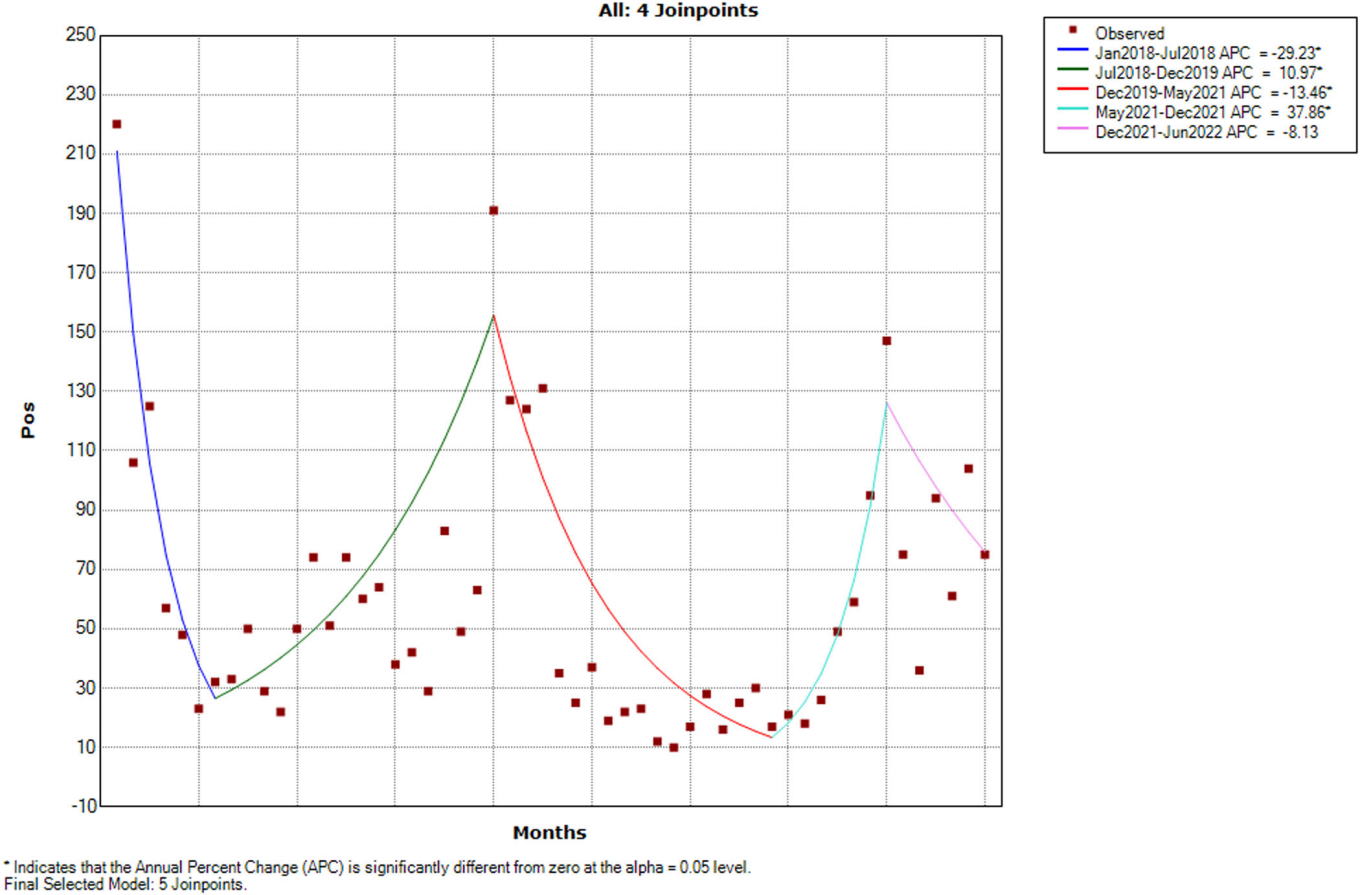
Joinpoint analysis of changes in respiratory viruses' positive cases per month.

Certainly, in light of the monthly‐based analysis, the joinpoint regression analysis identified four joinpoints (Figure 2). The ensuing APC values provided a quantitative understanding of the extent of these observed shifts within the monthly context.

Our analysis discerned a total of four distinct joinpoints, each indicative of specific monthly intervals:

1. From January 2018 to July 2018, our findings indicated a substantial and statistically significant reduction of positive cases by 29%. This initial period reflected a noteworthy decline in the occurrence of the variable monthly.
2. Moving forward to the period spanning July 2018 to December 2019, an opposite trend emerged, characterized by a statistically significant increasing pattern with an APC of 10.9%. During this interval, the variable demonstrated consistent monthly growth.
3. Subsequently, spanning December 2019 to May 2021, our analysis revealed a statistically significant decrease of positive cases by 13.46%. This interval marked a return to a declining monthly trend following the prior period of growth.
4. Shifting to the period between May 2021 and December 2021, a distinct change was observed, characterized by a statistically significant surge of 37.86% in positive cases monthly, indicating rapid growth.

Lastly, from December 2021 to July 2022, the APC value reflected a nonsignificant monthly decrease of 8.13%.

**Figure 3:**
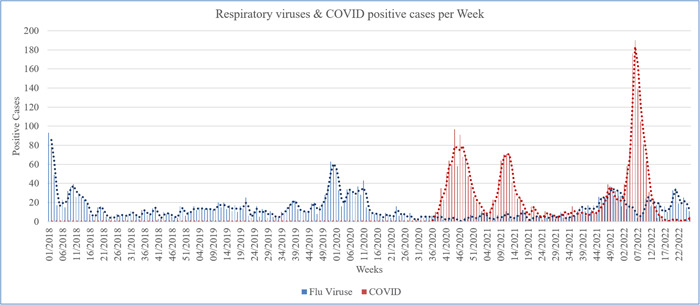
Weekly incidences of respiratory viruses in comparison to weekly incidences of COVID cases from 2018 to 2022.

The weekly numbers of respiratory virus cases detected before and after the start of the lockdown are illustrated in Figure 3. Following the lockdown's initiation (Week 11 2020), there was a discernible reduction in cases related to respiratory viruses. To further investigate the features of the respiratory viruses' epidemiology, our analysis focused on the time frame spanning from Week 40 2020 to Week 8 2021. This specific period typically corresponds to the peak of seasonal influenza activity during conventional years. Our analysis involved a comparative assessment, contrasting these results with those from the preceding two seasons (2018/2019 to 2019/2020).

Our findings indicate a substantial reduction in respiratory virus cases during this study period. Specifically, a 58%–82% reduction was detected in comparison to the respiratory virus activity observed during the previous two seasons (2018/2019 to 2019/2020). This marked decrease underscores a significant alteration in the prevalence of respiratory virus cases, signifying a distinct deviation from the expected seasonal epidemic pattern.

These results collectively underscore the influence of the lockdown measures on respiratory virus epidemiology. The observed reduction in cases postlockdown aligns with shifts in respiratory virus activity, hinting at the interplay between preventive measures and the observed changes in transmission dynamics. Moreover, the considerable reduction in influenza cases during a period of anticipated heightened activity underscores the potential role of external factors, possibly including altered behaviors and public health interventions, in reshaping the course of seasonal influenza outbreaks.

## 4 DISCUSSION

The mode of transmission of SARS‐CoV‐2 is similar to that of influenza. According to the World Health Organization, the basic measures for protecting against COVID‐19 are similar to those for influenza: frequent hand washing; social distancing; avoiding touching the eyes, nose, and mouth; and wearing a face mask when appropriate.^15^

Our results demonstrated a significant decrease in respiratory viruses, during the COVID‐19 pandemic, further, there was no annual laboratory‐confirmed respiratory virus outbreak or epidemic detected during the 2020 winter season. Infection control measures, including wearing masks, hand hygiene, and social distancing, may contribute not only to the prevention of COVID‐19 but also to decreases in the incidence of other viral infections and pneumonia. We postulate that lockdowns and border controls have markedly changed social behaviors, resulting in substantial reductions in contact between flu‐infected individuals and flu‐susceptible individuals.

During May 2021 to December 2021, a significantly increasing flu infection trend of 37.86% was observed at a time of increasing social contacts during reopening may have contributed to these findings. Likewise, in Hong Kong, after the reopening of schools and child‐care centers, a large number of outbreaks of acute upper respiratory tract infections occurred.^16^ Conversely, a study in Finland showed that the reopening of schools and daycare centers seems to have had no immediate impact on the incidence of any respiratory pathogens.^17^

Our data, presented here, is consistent with what was reported from southern hemisphere countries in the MMWR by the CDC,^10^ as well as data reported from the southern hemisphere from New Zealand, which showed a significant downward temporal trend in influenza virus detections during and post lockdown compared with prelockdown results.^18^ In Austria, they observed a rapid and statistically significant reduction of cumulative cases for all these viruses within a short time after the lockdown in March 2020, compared to previous seasons (each *p* < .001), while for the seasonally occurring respiratory viruses the lockdown led to the end of the annual epidemics.^19^

The World Health Organization European Region (which is a part of the northern hemisphere) reported a reduction in sentinel influenza virus‐positive detections between weeks 40/2020 and 8/2021 when compared to the same period in the previous six seasons.^20^ Chan et al. came to similar conclusions in the Northern Hemisphere, which represented a decrease in influenza seasonal duration, and a rapid descent within 7–12 weeks.^21^ Other research groups have already published similar results: In China, beginning in mid‐January 2020, influenza activity declined significantly, earlier than that of COVID‐19, they speculate that China's strict epidemic‐prevention measures against COVID‐19 resulted in a significant decline in laboratory‐confirmed influenza cases.^22^ In Korea during the period of enforced social distancing in 2020, research revealed that influenza hospitalization cases were 11.9–26.9‐fold lower compared with previous seasons, additionally, it showed that the national response strategies not only reduced SARS‐CoV‐2 cases but also substantially decreased influenza activity when compared with recent seasons.^23^

In Germany, compared to the two previous years, seasonal influenza and RSV incidence was eliminated during the COVID‐19 pandemic; they suggested that corona‐related measures and human behavior patterns could lead to a significant decline or even complete suppression of other respiratory viruses such as influenza and RSV.^24^ In Canada, a dramatically lower percentage of tests positive for all seasonal respiratory viruses during 2020–2021 compared to pre‐pandemic seasons was observed, with an absence of the annual seasonal epidemic of most seasonal respiratory viruses in 2020–2021.^25^ This decrease in the detection of respiratory viruses is consistent with a study from the United Kingdom that showed that the emergence of SARS‐CoV‐2 was associated with substantial reductions in the circulation of seasonal respiratory viruses.^26^ Evidence exists in the literature that supports the idea that personal protective measures (wearing masks, physical distancing, washing hands, and avoiding crowded places) with high feasibility could be implemented during influenza epidemics to reduce transmission.^11^

Overall, our findings are in line with an accumulated body of research indicating strict public health measures, such as regional lockdowns, border closures, hand washing, and facemask wearing may be effective in significantly reducing the spread of epidemic respiratory viruses. Another potential explanation for the decrease in seasonal respiratory viruses despite ongoing SARS‐CoV‐2 detection is a possible reduction in testing and/or laboratory reporting for non‐SARS‐CoV‐2 respiratory viruses. However, it is difficult at this time to determine whether there is also the potential impact of viral‐viral interactions between SARS‐CoV‐2 and other respiratory viruses contributing to the observed decline in circulation.

### 4.1 Study limitations

One limitation of this study is the potential for selection bias due to the retrospective nature of the chart review. This bias could affect the generalizability of the findings to a broader population and limit the ability to draw comprehensive conclusions about the overall impact of the SARS‐CoV‐2 pandemic and its interventions on respiratory virus transmission rates. In addition to the previously mentioned limitation, another important constraint of this study is the consideration of a specific population group (cancer patients), and this data consists mainly of hospital‐based data from a single medical center. Consequentially, the broader applicability of the findings to other cancer patient populations or demographic groups remains uncertain. A more detailed analysis of the cancer patient subgroup would enhance the study's ability to provide targeted recommendations and insights for healthcare practitioners working with this vulnerable population and would strengthen the overall robustness and relevance of the research. Finally, during the COVID‐19 pandemic, COVID‐19 testing was prioritized while respiratory virus testing was reduced; furthermore, the government set up several community‐based testing centers around the country to provide access to safe and free sampling for COVID‐19, which may result in selection bias.

## 5 CONCLUSION

Respiratory virus activity declined substantially, suggesting that the measures taken for COVID‐19 including wearing face masks, hand washing, school closures, reduced travel, increased ventilation of indoor spaces, and avoiding the 3Cs were effective in reducing the spread of other viral respiratory diseases. A reduction was observed in cumulative cases of respiratory viruses within a short time after the lockdown in March 2020, compared to previous seasons. Our findings suggest that interventions applied to control COVID‐19 may serve as useful strategies for the prevention and control of respiratory viruses in the upcoming seasons.

## AUTHOR CONTRIBUTIONS

Sawsan Mubarak contributed to the study's conception and design. Ala'a Hassan was responsible for data collection. Hadeel AlGhawrie contributed to the analysis of the data. Sawsan Mubarak, Osama Alsmadi, and Hadeel AlGhawrie contributed to the interpretation of data, took responsibility for the integrity of the data and the accuracy of the data analysis, and drafted the initial manuscript. All authors have reviewed and approved the final manuscript.

## CONFLICT OF INTEREST STATEMENT

The authors declare no conflict of interest.

## ETHICS STATEMENT

Ethical clearance was obtained from the Institutional Review Board Committee of King Hussein Cancer Center. All investigations were conducted in conformity with the ethical principles of research. Informed consent was waived by the Institutional Review Board Committee. The authors confirm that all methods were carried out following the relevant guidelines and regulations.

## Data Availability

The data used and analyzed during this study is included as supplementary information to this article. For further questions regarding the reuse of data, please contact the corresponding author (HA.15907@KHCC.JO).
